# Pharmacological sympathetic denervation prevents the development of polycystic ovarian syndrome in rats injected with estradiol valerate

**DOI:** 10.1186/s12958-018-0400-8

**Published:** 2018-09-07

**Authors:** Julieta A. Espinoza, Wendy Alvarado, Berenice Venegas, Roberto Domínguez, Leticia Morales-Ledesma

**Affiliations:** 1 Biology of Reproduction Research Unit, Physiology of Reproduction Laboratory, Facultad de Estudios Superiores Zaragoza, UNAM AP 9-020, CP 15000 México, DF Mexico; 2 Facultad de Ciencias Biológicas de la Benemérita Universidad Autónoma de Puebla, Blvd. Valsequillo, Av. San Claudio, Edificio 112-A, Cd Universitaria, Col. Jardines de San Manuel, Puebla, Mexico

**Keywords:** Polycystic ovary syndrome, Estradiol valerate, Innervation, Ovulation, Steroid hormones

## Abstract

**Background:**

The injection of estradiol valerate in female rats induces polycystic ovary syndrome, which is characterized by polycystic ovaries, anovulation, and hyperandrogenism. These characteristics have been associated with an increase in the ovarian concentration of norepinephrine, which occurs before establishing the polycystic ovary syndrome. The bilateral section of the superior ovarian nerve restores ovarian functions in animals with polycystic ovary syndrome. The superior ovarian nerve provides norepinephrine and vasoactive intestinal peptide to the ovary. An increase in the activity of both neurotransmitters has been associated with the development of polycystic ovary syndrome. The purpose of the present study was analyzed the participation of the noradrenergic nervous system in the development of polycystic ovary syndrome using guanethidine as a pharmacological tool that destroys peripheral noradrenergic nerve fibers.

**Methods:**

Fourteen-day old female rats of the CIIZ-V strain were injected with estradiol valerate or vehicle solution. Rats were randomly allotted to one of three guanethidine treatment groups for denervation: 1) guanethidine treatment at age 7 to 27-days, 2) guanethidine treatment at age 14 to 34- days, and 3) guanethidine treatment at age 70 to 90- days. All animals were sacrificed when presenting vaginal oestrus at age 90 to 94-days. The parameters analyzed were the number of ova shed by ovulating animals, the ovulation rate (i.e., the numbers of ovulating animals/the numbers of used animals), the serum concentration of progesterone, testosterone, oestradiol and the immunoreactivity for tyrosine hydroxylase enzyme. All data were analyzed statistically. A *p*-value of less than 0.05 was considered significant.

**Results:**

Our results show that the elimination of noradrenergic fibers before the establishment of polycystic ovary syndrome prevents two characteristics of the syndrome, blocking of ovulation and hyperandrogenism. We also found that in animals that have already developed polycystic ovary syndrome, sympathetic denervation restores ovulatory capacity, but it was not as efficient in reducing hyperandrogenism.

**Conclusion:**

The results of the present study suggest that the noradrenergic fibers play a stimulant role in the establishment of polycystic ovary syndrome.

## Background

Polycystic ovary syndrome (PCOS) is the most common endocrine disorder which affects between 6 and 10% of women in reproductive age [1, 2]. In 2006, the Androgen Excess & PCOS Society concluded that in the diagnosis of PCOS, the disorder predominantly consists of androgen excess and ovulatory dysfunction (i.e., chronic ovulatory dysfunction or polycystic ovarian morphology), with the exclusion of specific disorders, such as nonclassic adrenal 21-hydroxylase deficiency, Cushing’s syndrome, hyperprolactinemia, and androgen-producing tumors [3]. Frequently, PCOS is accompanied by obesity, hirsutism, and in the vast majority of cases, infertility [4]. In addition, some women with PCOS present metabolic alterations such as hyperinsulinemia and insulin resistance, the severity of both disorders increases in the presence of obesity [5] and the risk of cardiovascular diseases [6]. Some of the medical treatments for inducing ovulation in patients with the syndrome are: treatment with clomiphene citrate (an antiestrogen) [7], treatment with exogenous follicle stimulating hormone (FSH) [8], wedge resection of the ovaries [9] or treatment with low frequency electroacupuncture [10].

The etiology of PCOS is currently unknown. Evidence suggesting that the syndrome may originate in the hypothalamus, due to a primary neuroendocrine defect in gonadotropin-releasing hormone (GnRH) secretion that leads to increased frequency and amplitude in the pulses of the secretion of luteinizing hormone (LH) [11]. Recent studies suggests that ovarian innervation also plays a role in the physiopathology of the syndrome, since it has been observed that, in both, rodent with the induced pathology and women with the syndrome, there is an increase in sympathetic ovarian nervous activity [12–15].

In the rat, the ovary receives sympathetic innervation from two neural pathways: the ovarian plexus nerve (OPN) and the superior ovarian nerve (SON). The SON-fibers innervate the thecal cells and provide the ovary with norepinephrine (NE), mainly; but also, with neuropeptide Y (NPY) and vasoactive intestinal peptide (VIP) [16, 17]. In prepubertal rats, the bilateral section of the SON decreases ovarian NE concentrations at the first 24 h post-denervation [16, 18].

In prepubertal and adult rats, injection with 2 mg of estradiol valerate (EV), a long-acting estrogen, induces PCOS [19–22]. Rats with EV-induced PCOS are characterized by early puberty [21, 22], loss of estrous cycles [21, 23], high concentrations of androgens [22], anovulation, and around 60 days after EV injection, follicular cysts in the ovaries are observed [19]. The polycystic ovaries developed by the EV-injection show a reduction in their size, absence of corpora lutea, development of follicular cysts and poor follicular development [21, 24]. Because the structure of the ovaries of rats injected with EV is different from that of the ovary of women with PCOS, some authors have suggested having precautions when comparing these polycystic ovaries [25].

The development of PCOS has also been associated with changes in the catecholaminergic activity of the ovary. These changes include increase ovarian NE concentration and in the activity of tyrosine hydroxylase (TH), a limiting enzyme in NE biosynthesis, and a decrease in the number of beta-adrenergic receptors [12]. In this PCOS model, spontaneous ovulation occurs after the bilateral section of the SON and the androgens concentration in the ovary is normalized [20–22]. Parra et al. [26] showed that in addition to ovarian NE concentration increases, EV-treated rats also show an increase in VIP concentration, which could stimulate the onset of PCOS. This is possible, since ovaries of rats with PCOS cultivated in the presence of VIP release higher amounts of androgens and oestradiol than ovaries of rats without the syndrome.

Since surgically sectioning the SON of rats with PCOS does not allow us to distinguish whether the reestablishment of ovulation and serum androgen concentrations is due to a decrease in ovarian NE or VIP levels, the aim of the present study was to analyze the participation of the noradrenergic system in the onset of PCOS. For this purpose, we used guanethidine monosulfate (GTD) as a pharmacological tool that destroys only peripheral noradrenergic fibers [27], as GTD does not cross the blood-brain barrier [28]. GTD does not accumulate in other cell types (i.e. sensory neurons) nor does it cause their destruction [28, 29]. Our working hypothesis was that eliminating of noradrenergic fibers before or during PCOS onset would result in spontaneous ovulation and normal testosterone levels, while the same treatment after PCOS onset would reverse the ovulation blockage caused by the syndrome. In the present study, the treatment consisted of chronically injecting GTD for three weeks, before, during or after EV treatment to induce PCOS. Our data suggest that the noradrenergic fibers play a stimulant role in the establishment of PCOS.

## Methods

All experiments were carried out in strict accordance with the Mexican Law of Animal Treatment and Protection Guidelines, and the specifications of the Mexican Official Standard, NOM-062-ZOO-1999. The Committee of the Facultad de Estudios Superiores Zaragoza approved the experimental protocols. All efforts were taken to minimize the number of animals used, and all procedures were undertaken in a humane manner.

The study was performed using pre-pubertal female rats of the CIIZ-V strain from our own breeding stock. Animals were maintained under controlled lighting conditions (lights on from 05:00 to 19:00 h); with free access to food (Purina S.A., México) and tap water.

### Animal treatment

Following previously described methodologies [22, 30], PCOS was induced by injecting 14-day old rats with a single 2 mg EV (Sigma Chem. Co., St. Luis, Mo. USA) dose dissolved in 0.1 ml of sesame oil (Vehicle Vh, Sigma Chem. Co., St. Luis, Mo. USA). The control group consisted of 10 animals injected with Vh only.

**Fig. 1:**
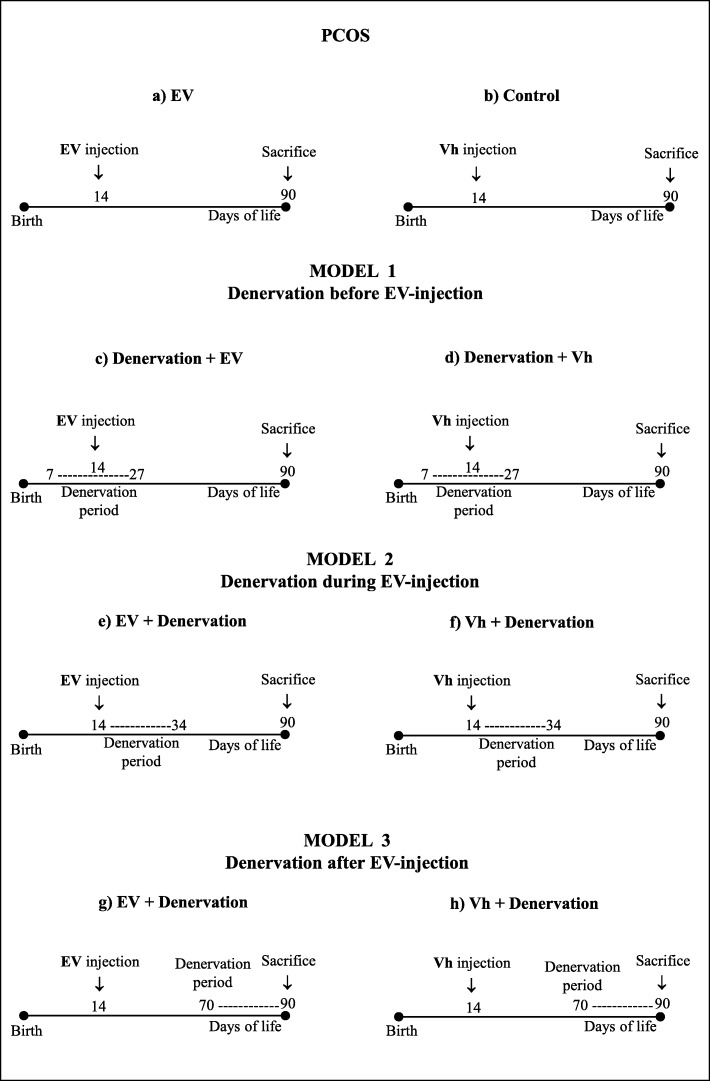
Experimental design showing the groups corresponding to the study models. In all cases, animals were sacrificed at 90–94 days of life, when the rats presented a vaginal oestrus

To analyze the effect that noradrenergic denervation has on PCOS onset, GTD denervation treatment was performed before, during and after EV-treatment to induce PCOS (Fig. 1).

Rats were randomly assigned to one of the following experimental groups described below.

#### Model 1. GTD denervated rats before EV treatment

Following the Lara model [31], to test whether the elimination of noradrenergic fibers prevents the onset of PCOS, 21 rats were denervated by chronic intraperitoneal chronic injection of 50 mg/kg GTD (Sigma Chem. Co., St. Luis, Mo. USA) for three weeks, from age 7 to 27-days old. At 14-days of age, 11 of the 21 rats treated with GTD were injected with EV, and the remaining 10 animals with Vh.

#### Model 2. Rats denervated with GTD at the same time of EV treatment

To assess if GTD denervation treatment prevents PCOS onset when initiated at the same time as the EV treatment, ten 14-day old rats were injected with EV and subsequently with the first three week dose of GTD, as described in the previous model, from age14 to 34-days old. The control group consisted of fourteen 14-day old animals injected with Vh and treated with GTD during the same (age14 to 34-days old) period.

#### Model 3. Rats with induced PCOS subsequently denervated with GTD

Since ovarian cysts develop 56 days after EV injection [19], this model served to analyze if denervation with GTD restores ovarian function in animals with an established PCOS. Twelve 14-day old animals were injected with EV and after 56 days with GTD for three weeks (from 70 to 90 days). The control group consisted of ten 14-day old animals treated with Vh and with GTD during the same (age70 to 90 days) period.

### Autopsy procedures

All animals were sacrificed when presenting vaginal oestrus, at 90 to 94 days of age. Eighty percent of the animals from each experimental group were sacrificed by decapitation and the remaining 20% with an overdose of sodium pentobarbital (PiSA Agropecuaria, S.A. de C.V.).

Animals sacrificed by decapitation: Sacrifice and autopsy procedures were performed between 10.00 AM and noon. Trunk blood was collected, allowed to clot, and centrifuged during 15 min at 3000 RPM. The serum was stored at − 20 °C until progesterone, testosterone and oestradiol concentrations were measured. During autopsy the oviducts were dissected and the number of ova was counted with the aid of a dissecting microscope (Nikon, Model C-PS). Ovaries were removed and processed for ovarian morphology analysis.

Animals sacrificed with sodium pentobarbital: An overdose of pentobarbital was injected at 10.00 time of day and perfused intracardially to corroborate GTD’s ability to destroy the noradrenergic fibers. This was carried out by the expression of TH in the ovaries, using the immunofluorescence technique.

The efficacy of GTD’s effects was assessed by measuring TH expression using the immunofluorescence technique.

### Analysis of ovarian morphology

To analyze the differences in ovarian morphology, three ovaries from each experimental group were cleaned of adherent fat tissue, fixed in Bouin solution for 24 h, dehydrated, embedded in paraplast, and serially sectioned at 10 μm. The sections were stained with hematoxylin-eosin and examined under a binocular microscope (Nikon, Model Labophot-2). All sections were analyzed for the presence of corpora lutea (CL), follicular cysts, and pre-cystic follicular structures. Following the criteria of Brawer et al. [19], follicular cysts were defined as follicles devoid of oocytes, displaying a large antral cavity, with an enlarged thecal cell layer, and a thin (most frequently monolayer) granulosa cell compartment containing apparently healthy cells. Pre-cystic follicles were defined as large follicles, with or without oocytes, containing four or five plicate layers of small, densely packed granulosa cells surrounding a very large antrum, and displaying a seemingly normal thecal compartment [19].

### Tyrosine hydroxylase immunofluorescence

The tyrosine hydroxylase immunofluorescence was performed following previously described methodology [32]. In brief, ovarian 8μm paraplast sections were mounted on coated glass slides. Each section was rinsed with PBS at pH 7.4, and twice with PBS-B (PBS with 0.5% Triton X-100). Sections were blocked by incubating them in IgG free 2% bovine serum albumin (BSA sigma) for 30 min to reduce background staining [33], and subsequently incubated at 4 °C overnight with a polyclonal rabbit anti-TH antibody (1:500) (Santa Cruz Biotechnology Inc., USA, sc-14,007). On the next day, slides were incubated with a secondary antibody (goat anti-rabbit FITC-labeled) (Vector Labs., CA, USA, FI-1000) for their visualization in the green channel. Slides were counterstained with Vectashield with DAPI (Vector Labs., CA, USA, H-1200). Sections of the ovaries were viewed and photographed using a Digital Camera (Nikon DS-U2, Japan) coupled with a fluorescence microscope (Nikon Eclipse E400, Japan). Intensity settings were kept constant between sections. The image analysis was performed using the Nis-Elements BR 3.0 system (Nikon). Immunofluorescence was calculated as the total number of pixels for the total area assessed by color segmentation analysis, which produces quantification by locating all objects of a specific color (green).

### Hormone measurement

Serum concentrations of progesterone (ng/ml), testosterone (pg/ml), and oestradiol (pg/ml) were measured using Enzyme-Linked Immunosorbent Assay (ELISA) techniques, with kits purchased from DRG Instruments GmbH (Marburg, Germany). The intra-and inter-assay coefficients of variation were 7.52% and 8.41% for progesterone, 6.42% and 7.32% for testosterone, and 8.5% and 9.3% for oestradiol, respectively. The standard curves for each hormone were in the following range: progesterone from 0 to 40 (ng/ml), testosterone from 0 to 16,000 (pg/ml) and oestradiol from 0 to 2000 (pg / ml). None of the samples indicated concentrations below the test’s sensitivity.

### Statistical analyses

Statistical analyses were performed using GraphPad InStant 3. The number of ova shed by ovulating animals was analyzed using a Kruskal-Wallis test, followed by a Mann-Whitney U-test. Ovulation rate (i.e., the numbers of ovulating animals/the numbers of used animals) was analyzed using a Fisher’s exact probability test. The fluorescence data and the serum concentration of progesterone, testosterone and oestradiol were analyzed with a two-tailed Student’s t test for comparing the results of two groups. A *p*-value of less than 0.05 was considered significant.

## Results

### Ovulatory response

None of the EV treated rats ovulated (0/10), while 10/10 of Vh-treated ones did. The number of ovulating animals and total number of ova shed was similar between Vh-treated and the GTD-treated (before, during or after) groups. No ovulation differences were observed between the Vh-treated control groups of rats and animals denervated with GTD before, during or after EV-treatment. When comparing the ovulatory response of EV-treated animals with GTD denervated animals we observed that ovulation was restored in the three study (EV-treatment before, during, or after GTD-treatment) groups (Table 1).

**Table 1:** Number of ovulating rats (numbers of ovulating animals/the numbers of used animals) and mean ± S.E.M. number of ova shed by rats injected with vehicle (Vh) or estradiol valerate (EV) at day 14 of life, and treated with GTD from 7 to 27 days of age (Model 1), or 14 to 34-day of age (Model 2) or at 70 to 90-day of age (Model 3). Rats were sacrificed in the adult stage at days 90–94 of life, on the day of vaginal oestrus

Groups	Number of ovulating rats		Total number of ova shed	
	Vh	EV	Vh	EV
Without denervation	10/10	0/10^a^	14.4 ± 0.7	0^c^
GTD (days 7–27)	9/10	8/11^b^	13.0 ± 1.3	11.2 ± 0.7 ^d^
GTD (days 14–34)	11/14	9/10 ^b^	13.2 0.7	10.6 ± 1.4 ^d^
GTD (days 70–90)	10/10	7/12^b^	10.3 ± 0.8	12.2 ± 0.8^d^

Number of ovulanting rats ^a^ *p* < 0.05 vs. group treated with Vh; ^b^ *p* < 0.05 vs. group treated with EV (Fisher’s exact probability test); total number of ova shed ^c^ *p* < 0.05 vs. group treated with Vh; ^d^ *p* < 0.05 vs. group treated with EV (Kruskal Wallis test, followed by Mann-Whitney U-test)

### Ovarian morphology

**Fig. 2:**
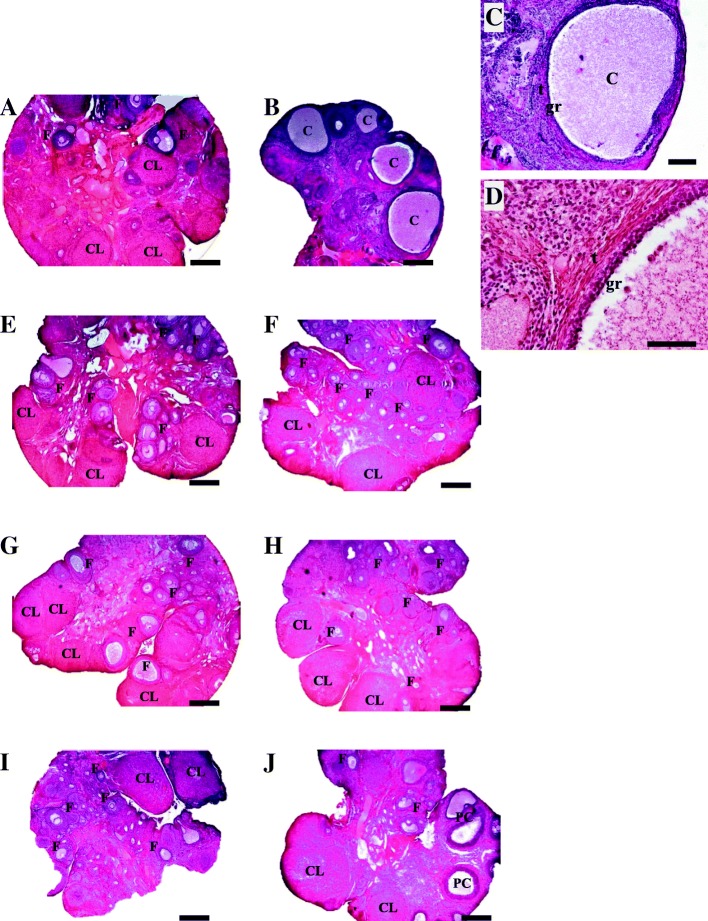
Ovarian histologies of the rats injected with **a** Vh or **b** EV at 14 days of age, **e** denervated with GTD (from days 7 to 27) + Vh at day 14 of life or **f** GTD (from days 7 to 27) + EV at day 14 of life, **g** Vh-injected at day 14 of life + GTD (from days 14 to 34) or **h** EV-injected at day 14 of life + GTD (from days 14 to 34), **i** Vh-injected at day 14 of life + GTD (from days 70 to 90) or **j** EV-injected at day 14 of life + GTD (from days 70 to 90). 4X microscopic lens, Scale Bar = 500 μm; **c** amplification of a cyst taken from Fig. B. 10X microscopic lens, Scale Bar = 200 μm; **d** thickening of the stratum of theca cells and thinning of the stratum of the granulosa cell of a follicular cyst taken from Fig. C. 40X microscopic lens, Scale Bar = 50 μm. Rats were sacrificed in the adult stage at days 90–94 of life, on the day of vaginal estrus; F: follicles in different stages of maturation; CL: corpora lutea; PC: pre-cyst; C: cyst; gr: granulosa cells; t: thecal cells

Ovaries from the Vh group appeared normal, and various primary and secondary follicles were apparent. Some of the follicles were atretic and showed nuclear pyknosis and some showed cell degeneration in the granulosa cell layer. Numerous corpora lutea were also observed (Fig. 2a).

The ovaries of EV-treated rats were small and showed very few follicles in development. Some of these follicles showed pyknosis of the granulosa cell layers. No large secondary or tertiary follicles, nor corpora lutea were observed (Fig. 2b). In addition, the ovaries of these rats showed numerous cysts which a very large central cavity (Fig. 2c), a highly attenuated granular layer and an abnormally thick theca layer (Fig. 2d).

In any of the three treatment periods studied, ovaries of rats treated with Vh and denervated with GTD showed a similar appearance to those of rats injected only with Vh (Fig. 2e, g, i). On the other hand, ovaries of animals injected with EV and denervated with GTD exhibited follicles at various stages of development as well as corpora lutea, observations that confirm ovulation (Figs. 2f, h, j). In addition, no follicular cysts were observed in the ovaries of rats treated with GTD before or immediately after EV-treatment. In contrast, some cystic structures were visible in animals denervated 56 days after EV-treatment (Fig. 2j).

### Tyrosine hydroxylase expression in the ovary

**Fig. 3:**
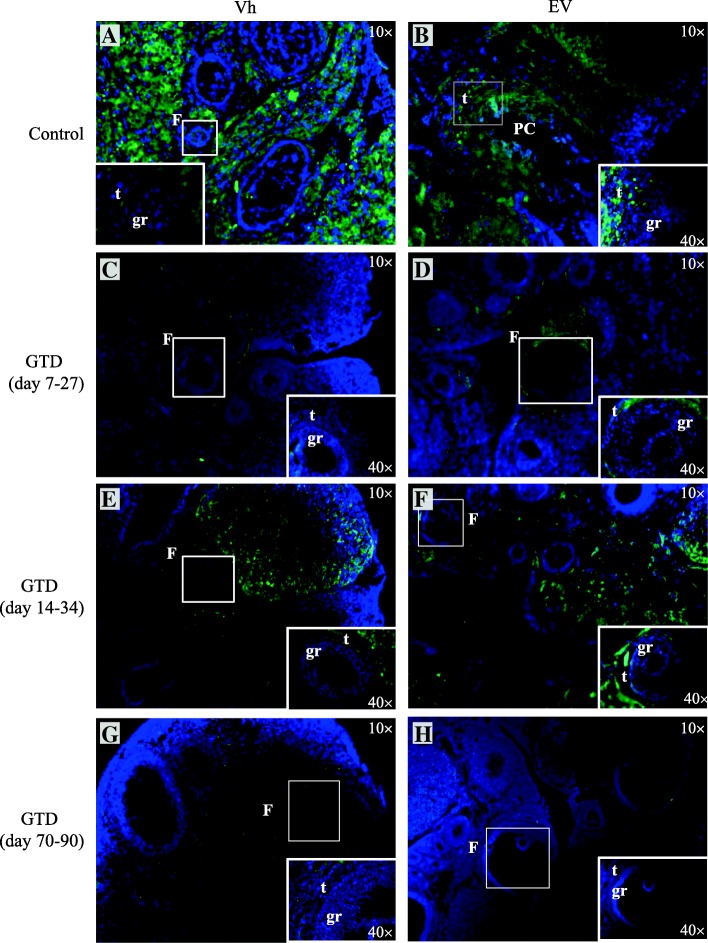
Immunofluorescence staining of tyrosine hydroxylase (TH) in ovaries of rats injected with **a** Vh or **b** EV at 14 days of age, **c** denervated with GTD (from days 7 to 27) + Vh at day 14 of life or **d** GTD (from days 7 to 27) + EV at day 14 of life, **e** Vh-injected at day 14 of life + GTD (from days 14 to 34) or **f** EV-injected at day 14 of life + GTD (from days 14 to 34), **g** Vh-injected at day 14 of life + GTD (from days 70 to 90) or **h** EV-injected at day 14 of life + GTD (from days 70 to 90). Ovarian sections were stained with anti-TH antibody (green color), and nuclear staining with DAPI (blue color). F: follicles in different stages of maturation; PC: pre-cyst; gr: granulosa cells; t: thecal cells

**Fig. 4:**
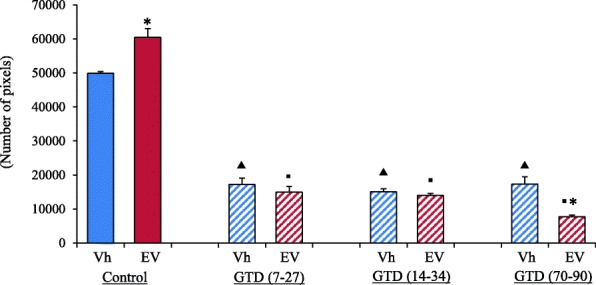
Analysis of the distribution of TH in ovaries of control-rats injected with Vh (blue bars) or EV (red bars) at 14 days of age; rats denervated with GTD (from days 7 to 27) + Vh (striped blue bars) or GTD (from days 7 to 27) + EV (striped red bars); Vh + + GTD (from days 14 to 34) (striped blue bars) or EV + GTD (from days 14 to 34) (striped red bars); Vh + GTD (from days 70 to 90) (striped blue bars) or EV + GTD (from days 70 to 90) (striped red bars). All animals were sacrificed at days 90–94 of life. The image analysis was performed using the Nis-Elements BR 3.0 system. *p < 0.05 versus immediate control group; ^▲^*p* < 0.05 versus Vh-control group; ^▪^*p* < 0.05 versus EV-control group (Student’s t-test)

As shown in Fig. 3, TH immunoreactivity was detected in the ovaries of both, the Vh-treated control group and the EV-treated group (Fig. 3a, b). Image analysis using the Nis-Elements BR system showed a greater TH expression in the EV-treated group than in the Vh-treated control group (Fig. 4). In both groups, the TH expression were observed around the follicles and in the interstitial gland. In the EV-treated groups, the TH expression were observed in the pre-cystic follicles. GTD-treatment before, during or after EV-treatment yielded lower ovarian TH expression (Fig. 3d, f, h). A similar effect was observed in the Vh-treated groups (Fig. 3c, e, g). A semiquantitative analysis of the images confirmed that, compared to the Vh-treated control group, animals in all GTD- treated groups showed lower TH expression. Compared to the EV only or Vh only treatment groups, all GTD-treated groups showed lower TH expression (Fig. 4).

### Hormone concentrations in serum

**Fig. 5:**
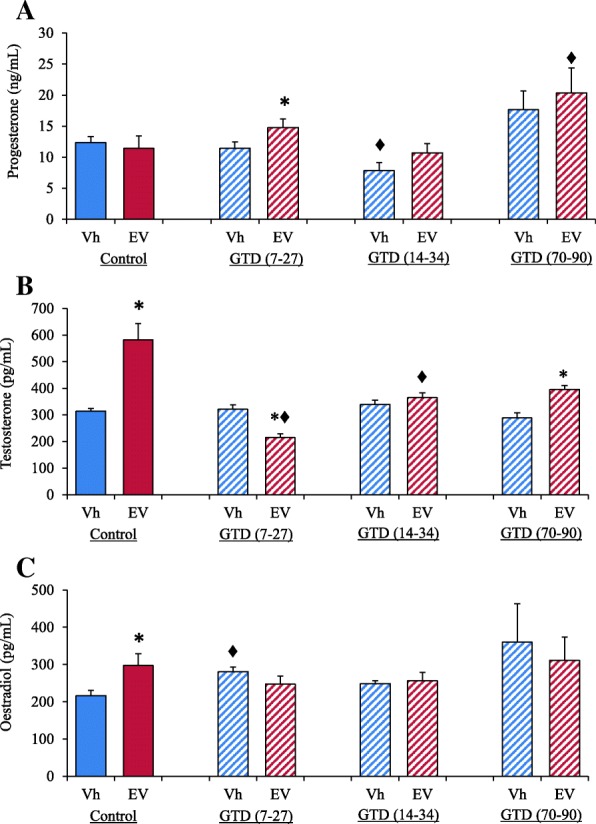
Mean ± S.E.M. progesterone (ng/ml) (**a**), testosterone (pg/ml) (**b**) and estradiol (pg/ml) (**c**) serum levels in control-rats injected with Vh (blue bars) or with EV (red bars) at 14 days of age; rats denervated with GTD (from days 7 to 27) + Vh (striped blue bars) or GTD (from days 7 to 27) + EV (striped red bars); Vh + + GTD (from days 14 to 34) (striped blue bars) or EV + GTD (from days 14 to 34) (striped red bars); Vh + GTD (from days 70 to 90) (striped blue bars) or EV + GTD (from days 70 to 90) (striped red bars). All animals were sacrificed at days 90–94 of life when the rats presented a vaginal oestrus. *p < 0.05 versus immediate control group; ♦*p* < 0.05 versus corresponding control group without GTD (Student’s t-test)

Compared to the Vh-treatment control group, EV-treatment did not modify progesterone levels in serum. In animals Vh-treated and GTD-treated at 14 to 34-day of age progesterone levels were lower than in the Vh-treated control group, while in the other periods of denervation was not modified. In the 7 to 27-days of age groups, EV-treated rats showed higher progesterone levels than Vh-treated group. Compared to the EV-only treatment group, rats subsequently treated with GTD from 14 to 34 days of age did not show changes in progesterone concentration, while rats with denervation treatment at 70 to 90-days of age showed higher progesterone levels (Fig. 5a).

Compared to the Vh-treatment control group, EV-treated animals showed higher testosterone concentrations, while, no testosterone concentration changes were observed in the GTD-treatment groups (Study Model 1, 2 or 3). Compared to the EV-treated group, testosterone levels were lower in rats EV-treated and GTD-treatment at 7 to 27-days of age (Model 1) and at 14 to 34-day of age (Model 2). EV-treated animals denervated at 70 to 90 days of age showed higher testosterone concentrations than the Vh-treated and GTD from 70 to 90 days of age (Fig. 5b).

Compared to the Vh-treatment control group, higher estradiol levels were measured in EV-treated rats and in the Vh-treated and GTD-treatment at 7 to 27-days of age. The same treatment on the other two age groups did not have an apparent effect on estradiol levels (Fig. 5c).

## Discussion

Taken together, the results of the present study shows that PCOS onset in pre-pubertal EV-treated rats depends on the integrity of the ovary’s peripheral noradrenergic system.

The ovary is innervated by sympathetic fibers through the SON and OPN. In addition to containing catecholamines synthetizing fibers, the SON are formed by fibers that synthesize other peptides; such as VIP and NPY; the supply of substance P (SP) and calcitonin gene-related peptide (CGRP) to the ovaries by the OPN [34]. The surgical section of these nerves has been used to analyze the role of innervation in ovarian functions. A research model to target specific types of neurons consist of treatments with neurotoxins, such as GTD; which when administered chronically and in high doses produce sympathectomy at the peripheral level [31, 35–37].

Studies with male rats by Heath and Burnstock, [38] show that chronic high-doses GTD treatment has no apparent effect on parasympathetic cholinergic neurons or sensory neurons. According to Angeletti et al. [39], injecting three doses of GTD decreases the number of catecholaminergic neurons in the superior cervical ganglion. Lara et al. [31] showed that in the pubertal rat, the chronic injection of GTD destroys the ovary’s catecholaminergic neurons. Tyrosine hydroxylase is the limiting enzyme in catecholamines biosynthesis, and is widely used as a norepinephrine biosynthesis marker [31]. An increase in TH activity and expression in the ovary has been documented in rats with EV-induced PCOS [12, 24, 40]. Our results support these findings, since the ovaries of EV-treated rats showed as increase in TH expression. Tyrosine hydroxylase expression was almost completely lost in GTD-treated animals before, during, or after EV-treatment. This result reinforces the accounts of a GTD-induced reduction of catecholaminergic neurons and agrees with the observations presented by Angeletti et al. [39] and Lara et al. [31].

To date, despite the exhaustive research on the etiology of PCOS, the causes of the syndrome are still unknown. One of the hypotheses proposed to explain the origin of PCOS suggests that increases in ovarian sympathetic nerve activity results in abnormal ovarian function [12, 13, 20, 24]. In women with PCOS, the ovaries show greater density of nerve fibers [14] and higher sympathetic nerve activity [41]. In EV-treated rats, and in rats exposed to chronic cold stress, the onset of PCOS is accompanied by increases in sympathetic nerve activity [13, 42]. In the EV-induced PCOS model, the surgical sectioning of the SON restores estral cyclicity, ovulatory capacity, and the presence of corpora lutea [20, 21]. This response has been explained by decreases in adrenergic tone; however, as previously described, in addition to providing adrenergic fibers to the ovary the SON is also a source of other peptides such as VIP.

Hence the importance of using a more specific tool, such as GTD, that only destroys catecholaminergic neurons [37]. In the present study, the results obtained from estimating TH expression in control and EV-treated animals with GTD denervation shows that regardless of the age of treatment, the number of pixels with the immunoreactive mark was significantly lower in GTD treated animals. These results confirm the success of catecholaminergic fibers denervation.

According to the criteria established in 2006 by the Society of Excess of Androgens and Polycystic Ovarian Syndrome, hyperandrogenism is the most important parameter in PCOS diagnosis. In EV-induced PCOS rats, hyperandrogenism is explained by increases in steroidogenic activity in theca cells of cystic and pre-cystic structures in the ovaries [43] and increases in NE concentrations [13].

This hypothesis is supported by present study results, by the time the rats reached adulthood EV-treated animals with chronic GTD treatment stopped PCOS onset (anovulation and high of testosterone concentrations).

According to Lara et al. [24], an increase in ovarian neural growth factor (NGF) concentration is observed 15 days after EV treatment, resulting in higher TH activity in the superior mesenteric celiac ganglion (CSMG) and the subsequent release of NE to the ovary that precedes development of ovarian cysts.

In models 1 and 2 of the present study, denervation with GTD began before the ovarian NGF increases, suggesting that TH activity increases was not present. We can’t rule out that PCOS onset had taken place and was resolved by the nerves inactivity. New experiments are necessary to evaluate such possibility.

In animals of Model 3, in which EV-induced PCOS is fully established, denervation of the noradrenergic fibers reaching the ovary is efficient at restoring ovulation, but the testosterone concentration decrease was not significant. This can be explained by the persistence of some pre-cystic structures in the ovaries of these rats. The persistence of pre-cystic structures in the ovaries in animals from Model 3 could be explained by the short time between denervation and autopsy. Unlike the animals in Model 1 and 2, where autopsy was performed at least 50 days after denervation treatment, animals in Model 3 were sacrificed immediately after denervation treatment, and the time elapsed between denervation treatment and autopsy was not enough to allow the remodeling of the ovarian structure.

In addition to preventing hyperandrogenism and anovulation, peripheral noradrenergic denervation in EV-treated animals prevented morphological alterations of the ovaries, a characteristic of animals with PCOS. The ovaries of these animals showed corpora lutea and follicles in different growth stages, which allow us to suggest that the animals would probably be able to ovulate in later cycles.

In mammals, the sympathetic innervation of the ovary stimulates ovulation and secretion of steroid hormones [18, 44, 45]. According to several authors, the role of the ovarian sympathetic innervation varies according to the animal’s endocrine stage [16, 17, 31, 46, 47].

In the present study, the results obtained from Vh-treated and GTD-denervated animals support this idea. Uchida and Kagitani [48] reported that the electrical stimulation of the SON results in lower testosterone concentrations via the activation of alpha-1 adrenergic receptors, and in lower oestradiol levels by the activation of alpha-2 adrenergic receptors. The discrepancy between Uchida and Kagitani’s findings and the present study results on testosterone and oestradiol secretion regulation in the Vh-treated and GTD denervated animals may result from the destruction of noradrenergic neurons by GTD treatment, evidenced by the TH (activity / expression) reduction.

According to Kuncová et al. [49], GTD-treatment to pre-pubertal rats does not modify immunoreactivity to VIP in cardiac tissue. According to Benarroch et al. [50], adults rats treated with GTD for 5 weeks showed increased immunoreactivity to VIP neurons in the CSMG and not to VIP neurons in the superior cervical ganglion. The authors indicate their results were due to differences in afferent inputs to prevertebral and paravertebral ganglia. Working with adult rats, we’ve previously shown [51] that the effects on progesterone, testosterone, and oestradiol secretion resulting from unilaterally injecting VIP into the ovary’s intra-bursal depends on the injected ovary, the day of the estrous cycle treatment was performed, and the time elapsed between treatment and autopsy. These results suggest that the asymmetric response of the ovaries to VIP treatment is modulated, at least in part, by the innervation received by each ovary [51]. The effects of VIP on steroid hormones release by the ovaries are modulated by neural signals arriving through the SON (NE and/or NPY) ([52], in press). According to Lara et al. [31] there is no information on whether VIP-containing nerves in the SON or VIP-producing ovarian cells remain functional in GTD-treated rats. Therefore, in the present study we cannot rule out the possibility that the GTD-treatment had an impact on the VIP effects on the ovaries.

Studies have shown that VIP is a potent stimulant of steroid hormones secretion, and its effects have been explained through the regulation of enzymes involved in steroidogenesis [53]. The effect that VIP has on regulating steroid hormone secretion has been shown in experimental models, where the ovaries of adult EV-treated rats were removed and placed in a culture medium with VIP can release a greater quantity of androgens and estrogens than the ovaries of EV-treated rats cultured without the VIP-stimulus [26].

## Conclusions

Taken together, the results of the present study support the notion that the increased peripheral noradrenergic tone seen in animals with EV-induced PCOS is an important factor in the onset of the pathophysiology.

There is evidence showing that even though the concentration of NE is high in rats with EV-induced PCOS, the reestablishment of ovarian function is not only due to a decrease in noradrenergic activity since ovulation restoration occurs in the innervated ovary and not in the denervated ovary of animals with EV-induced PCOS and unilateral sectioning of the SON [22].

Furthermore, in rats with EV-induced PCOS ovulation reestablishment occurs after unilateral or bilateral sectioning of the vagus nerve [30]. Morales-Ledesma et al. [54] showed that development the PCOS induced by subcutaneous injecting testosterone propionate was not modified by the unilateral or bilateral sectioning of the SON, suggesting that the etiology of PCOS depends not only on the hyperactivity of NE fibers and that other neurotransmitters are participating in the onset and maintenance of PCOS.

The present study contributes to the set of evidence that support the participation of the sympathetic innervation in PCOS onset and could lay the foundations to propose therapeutic alternatives that improve PCOS alterations. There is evidence that some women with PCOS can ovulate after treatment with low frequency electroacupuncture applied at the ovarian innervation origin level [55]. In clinical studies, low GTD doses have been used to reduce hypertension [56]. Since some patients with PCOS present metabolic alterations, including high blood pressure [57, 58], GTD treatment could reduce sympathetic activity and improve some of the alterations related to the PCOS. More studies are necessary to support such possibility.

## Abbreviations

CGRP: Calcitonin gene-related peptide
CL: Corpora lutea
CSMG: Superior mesenteric celiac ganglion
EV: Estradiol valerate
FSH: Follicle stimulating hormone
GnRH: Gonadotropin-releasing hormone
GTD: Guanethidine monosulfate
LH: Luteinizing hormone
NE: Norepinephrine
NPY: Neuropeptide Y
OPN: Ovarian plexus nerve
PCOS: Polycystic ovary syndrome
SON: Superior ovarian nerve
SP: Substance P
TH: Tyrosine hydroxylase
Vh: Vehicle
VIP: Vasoactive intestinal peptide

## References

[1] Homburg R (2007). Polycystic ovary syndrome. Best Pract Res Clin Obstet Gynaecol.

[2] Franks S (2008). Polycystic ovary syndrome in adolescents. Int J Obes.

[3] Lizneva D, Suturina L, Walker W, Brakta S, Gavrilova-Jordan L, Azziz R (2016). Criteria, prevalence, and phenotypes of polycystic ovary syndrome. Fertil Steril.

[4] Franks S (1995). Polycystic ovary syndrome. Med Prog.

[5] Morales AJ, Laughlin GA, Bützow T (1996). Insulin, somatotropic, and LH axes in lean and obese women with polycystic ovay syndrome: common and distinct features. J Clin Endocrinol Metab.

[6] Vrbikova J, Cifkova R, Jirkovska A, Lanska V, Platilova H, Zamrazil V, Starka L (2003). Cardiovascular risk factors in young Czech females with polycystic ovary syndrome. Hum Reprod.

[7] Hull MGR, Tepleton AA, Drife JO (1992). The causes of infertility and relative effectiveness of treatment. Infertility.

[8] Wang CF, Gemzell C (1980). The use of human gonadotropins for induction of ovulation in women with polycystic ovarian disease. Fertil Steril.

[9] Adashi EY, Rock JA, Guzick D (1981). Fertility following bilateral ovarian wedge resection: a critical analysis of 90 consecutive cases of the polycystic ovary syndrome. Fertil Steril.

[10] Stener-Victorin E, Bedel E, Manneras L (2008). Acupunture in polycystic ovary syndrome: current experimental and clinical evidence. J Neuroendocrinol.

[11] Taylor AE, Mccourt B, Martin KA (1997). Determinants of abnormal gonadotropin secretion in clinically defined women with polycystic ovary syndrome. J Clin Endocrinol Metab.

[12] Lara HE, Ferruz J, Luza S, Bustamante DA, Borges Y, Ojeda SR (1993). Activation of ovarian sympathetic nerves in polycystic ovary syndrome. Endocrinology.

[13] Lara HE, Dorfman M, Venegas M (2002). Changes in sympathetic nerve activity of the mammalian ovary during a normal estrous cycle and in polycystic ovary syndrome: studies on norepinephrine release. Microsc Res Tech.

[14] Heider U, Pedal I, Spanel-Borowski K (2001). Increase in nerve fibers and loss of mast cells in polycystic and postmenopausal ovaries. Fertil Steril.

[15] Luna SL (2012). In vivo beta-adrenergic blockade by propranolol prevents isoproterenol-induced polycystic ovary in adult rats. Horm Metab Res.

[16] Aguado LI, Ojeda SR (1984). Prepubertal ovarian function is finely regulated by direct adrenergic influences. Role of noradrenergic innervation. Endocrinology.

[17] Forneris LM, Aguado L (2002). Neonatal superior ovarian nerve transection disturbs the cyclic activity of the female rats. J Steroid Biochem Mol Biol.

[18] Chávez R, Carrizosa L, Domínguez R (1991). Effects of superior ovarian nerve on spontaneous and induced ovulation in adult rats. Med Sci Res.

[19] Brawer JR, Munoz J, Farookhi R (1986). Development of the polycystic ovarian condition (PCO) in the estradiol valerate-treated rat. Biol Reprod.

[20] Barria A, Leyton V, Ojeda SR, Lara HE (1993). Ovarian steroidal response to gonadotropins and β-adrenergic stimulation is enhanced in polycystic ovary syndrome: role of sympathetic innervation. Endocrinology.

[21] Rosa-E-Silva A, Guimares MA, Padmanabhan V, Lara HE (2003). Prepubertad administration of estradiol valerate disrupts cyclicity and leads to cystic ovarian morphology during adult life in the rat: role of sympathetic inervation. Endocrinology.

[22] Morales-Ledesma L, Linares R, Rosas G (2010). Unilateral sectioning of the superior ovarian nerve of rats with polycystic ovarian syndrome restores ovulation in the innervated ovary. Reprod Biol Endocrinol.

[23] Sotomayor-Zárate R, Dorfman M, Paredes A, Lara HE (2008). Neonatal exposure to estradiol valerate programs ovarian sympathetic innervation and follicular development in the adult rat. Biol Reprod.

[24] Lara HE, Dissen GA, Leyton V (2000). An increased intraovarian synthesis of nerve growth factor and its low affinity receptor is a principal component of steroid induced polycystic ovary in the rat. Endocrinology.

[25] Lansdown A, Rees A (2012). The sympathetic nervous system in polycycstic ovary syndrome: a novel therapeutic target?. Clin Endocrinil.

[26] Parra C, Fiedler JL, Luna SL, Greiner M, Padmanabhan V, Lara HE (2007). Participation of vasoactive intestinal polypeptide in ovarian steroids production during the rat estrous cycle and in the development of estradiol valerate-induced polycystic ovary. Soc Reprod Fertil.

[27] Manning PT, Powers CW, Schmidt RE, Johnson EMJ (1983). Guanethidine-induced destruction of peripheral sympathetic neurons occurs by an immune-mediated mechanism. J Neurosci.

[28] Johnson EM, Manning PT (1984). Guanethidine-induced destruction of sympathetic neurons. Int Rev Neurobiol.

[29] Jensen-Holm J, Juul P (1971). Ultrastructural changes in the rat superior cervical ganglion following prolonged guanethidine administration. Acta Pharmacol Toxicol.

[30] Linares R, Hernández D, Morán C (2013). Unilateral or bilateral vagotomy induces ovulation in both ovaries of rats with polycystic ovarian syndrome. Reprod Biol Endocrinol.

[31] Lara HE, McDonald JK, Ojeda SR (1990). Guanethidine-mediated destruction of ovarian sympathetic nerves disrupts ovarian development and function. Endocrinology.

[32] Venegas-Meneses B, Padilla JF, Juárez CE (2015). Effects of ovarian dopaminergic receptors on ovulation. Endocrine.

[33] Díaz A, De Jesús L, Mendieta L (2010). The amyloid-β25–35 injection into the CA1 region of the neonatal rat hippocampus impairs the long-term memory because of an increase of nitric oxide. Neurosci Lett.

[34] Dissen GA, Ojeda SR (1999). Ovarian Innervation. Enc Reprod.

[35] Burnstock G, Evans B, Gannon BJ, Heath JW, James V (1971). A new method of destroying adrenergic nerves in adult animals using guanethidine. Br J Pharmac.

[36] Heath JW, Evan BK, Gannon BJ, Burnstock G, James VB (1972). Degeneration of adrenergic neurons following guanethidine treatment: an ultrastructural study. Virchows Archly Abteilung B-Zellpathologie.

[37] Eränkö L, Eränkö O (1971). Effect of guanethidine on nerve cells and small intensely fluorescent cells in sympathetic ganglia of newborn and adult rats. Acta Pharmacol Toxicol.

[38] Heath JW, Burnstock G (1977). Selectivity of neuronal degeneration produced by chronic guanethidine treatment. J Neurocytol.

[39] Angeletti PU, Levi-Montalcini R, Caramia F (1972). Structural and ultrastructural changes in developing sympathetic ganglia induced by guanethidine. Brain Res.

[40] Manni L, Holmäng A, Lundeberg T, Aloe L, Stener-Victorin E (2005). Ovarian expression of alpha (1)- and beta (2)-adrenoceptors and p75 neurotrophin receptors in rats with steroid-induced polycystic ovaries. Auton Neurosci Basic Clin.

[41] Sverrisdottir YB, Mogren T, Kataoka J, Janson PO (2008). Stener-Victorin E. Is polycystic ovary syndrome associated with high sympathetic nerve activity and size at birth?. Am J Physiol Endocrinol Metab.

[42] Bernuci MP, Szawka RE, Helena CV, Leite CM, Lara HE, Anselmo Franci JA (2008). Locus Coeruleus mediates cold stress-induced polycystic ovary in rats. Endocrinology.

[43] Yen SSC, Yen SSC, Vela P, Rankin J (2001). Síndrome del ovario poliquístico (Anovulación crónica hiperandrogénica). Médica Panamericana S. A. Buenos Aires.

[44] Dyer CA, Erickson GF (1985). Norepinephrine amplifies human chorionic gonadotropin-stimulated androgen biosynthesis by ovarian theca-interstitial cells. Endocrinology.

[45] Morales L, Chávez R, Domínguez R (1993). Participation of the superior ovarian nerve in the regulation of ovulation in the prepuberal rat: differential effects of unilateral and bilateral section of the nerve. Med Sci Res.

[46] Flores A, Ayala ME, Domínguez R (1990). Does noradrenergic peripheral innervation have a different role in the regulation of ovulation in the prepubertal and the adult rat?. Med Sci Res.

[47] Morales-Ledesma L, Vieyra E, Ramírez D (2012). Effects on steroid hormones secretion resulting from the acute stimulation of sectioning the superior ovarian nerve to pre-puberal rats. Reprod Biol Endocrinol.

[48] Uchida S, Kagitani F (2015). Autonomic nervous regulation of ovarian function by noxious somatic afferent stimulation. J Physiol Sci.

[49] Kuncová J, Slavíková J, Reischig J (2003). Distribution of vasoactive intestinal polypeptide in the rat heart: effect of guanethidine and capsaicin. Ann Anat.

[50] Benarroch EE, Zollman PJ, Smithson IL, Schmelzer JD, Low PA (1994). Different reinnervation patterns in the celiac/mesenteric and superior cervical ganglia following guanethidine sympathectomy in adult rat. Brain Res.

[51] Rosas G, Ramírez MI, Linares R, Trujillo A, Domínguez R, Morales-Ledesma L (2015). Asymmetric steroidogenic response by the ovaries to the vasoactive intestinal peptide. Endocrine.

[52] Rosas G, Linares R, Ramírez DA, Vieyra E, Trujillo A, Domínguez R, Morales-Ledesma L (2018). The neural signals of the superior ovarian nerve modulate in an asymmetric way the ovarian steroidogenic response to vasoactive intestinal peptide. Front Physiol.

[53] Davoren JB, Hsueh AJW (1985). Vasoactive intestinal peptide: a novel stimulator of steroidogenesis by cultured rat granulosa cells. Biol Reprod.

[54] Morales-Ledesma L, Díaz Ramos JA, Hérnández Trujillo A (2017). Polycystic ovary syndrome induced by exposure to testosterone propionate and effects of sympathectomy on the persistence of the syndrome. Reprod Biol Endocrinol.

[55] Stener-Victorin E, Waldenström U, Tägnfors U, Lundeberg T, Lindstedt G, Janson PO (2000). Effects of electro-acupuncture on anovulation in women with polycystic ovary syndrome. Acta Obstet Gynecol Scand.

[56] Abad-Santos F, Novalbos J, García AG, Lorenzo- Velázquez B, Lorenzo P, Moreno A (2008). Sístema nervioso simpático: fármacos simpaticolíticos. Médica Panamericana S. A. Buenos Aires.

[57] Tekin G, Tekin A, Kilicarslan EB, Haydardedeoglu B (2008). Altered autonomic neural control of the cardiovascular system in patients with polycystic ovary syndrome. Int J Cardiol.

[58] Ehrmann DA, Liljenquist DR, Kasza K, Azziz R, Legro RS, Ghazzi MN (2006). Prevalence and predictors of the metabolic syndrome in women with polycystic ovary syndrome. J Clin Endocrinol Metab.

